# Rapid Dissolution of Noble Metals in Organic Solvents

**DOI:** 10.1002/cssc.202201285

**Published:** 2022-09-01

**Authors:** Abhijit Nag, Carole A. Morrison, Jason B. Love

**Affiliations:** ^1^ EaStCHEM School of Chemistry University of Edinburgh Edinburgh EH9 3FJ United Kingdom

**Keywords:** electronic waste, gold dissolution, noble metals, recycling, sustainable chemistry

## Abstract

The dissolution of elemental noble metals (NMs) such as gold, platinum, palladium, and copper is necessary for their recycling but carries a high environmental burden due to the use of strong acids and toxic reagents. Herein, a new approach was developed for the rapid dissolution of elemental NMs in organic solvents using mixtures of triphenylphosphine dichloride or oxalyl chloride and hydrogen peroxide, forming metal chloride salts directly. Almost quantitative dissolution of metallic Au, Pd, and Cu was observed within minutes at room temperature. For Pt, dissolution was achieved, albeit more slowly, using the chlorinating oxidant alone but was inhibited on addition of hydrogen peroxide. After leaching, transfer of Pt^IV^ and Pd^II^ chloride salts from the organic phase into a 6 m HCl aqueous phase facilitated their separation by precipitation of Pt^IV^ using a simple diamide ligand. In contrast, the retention of Au^III^ chloridometalate in the organic phase allowed its selective separation from Ni and Cu from a leachate solution obtained from electronic CPUs. This new approach has potential application in the hydrometallurgical leaching and purification of NMs from ores, spent catalysts, and electronic and nano‐wastes.

## Graphical abstract

**Leaching**: Noble metals such as Au, Pd, Pt, and Cu are leached rapidly in organic solvents using the cheap and recyclable chemicals triphenylphosphinedichloride/oxalyl chloride, and the green oxidant hydrogen peroxide. This procedure could replace classical and highly toxic leaching methods for e‐waste recycling.

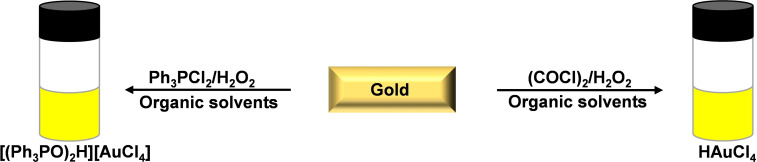

## Introduction

Noble metals (NMs) such as gold (Au), palladium (Pd), platinum (Pt) are well‐known for their uses in jewellery, investment, modern electronics, medicine, and chemical catalysis. They are extracted from ores with very low abundance and, as such, the “mining” of secondary resources such as electronic waste (e‐waste) has become an increasingly attractive proposition due to the higher abundance of NMs in these materials compared with mining deposits.[^1^, ^2^] The recycling of NMs from e‐waste would improve metal sustainability and enhance the circularization of modern technological processes.^[1]^

Hydrometallurgical refining and recycling processes generally comprise leaching of metals from the ore or secondary source, separation of the selected metal ions from the mixed‐metal leachate, and isolation/reduction of the separated metal ions as the elemental metal or metal compound. However, the individual steps within these processes are often associated with high energy consumption and environmental burden due to the nature of the chemical reagents employed. As such, many new approaches have been recognized, particularly those that improve the metal separation step. For example, Stoddart and co‐workers exploited host–guest supramolecular strategies to precipitate selectively NMs such as gold and platinum using α‐cyclodextrins and cucurbiturils.[^3^, ^4^, ^5^] Yavuz and co‐workers used porphyrin‐based polymers to extract selectively NMs from e‐waste.^[6]^ Liu et al. reported a method in which different types of macrocyclic tetralactam receptors were used to form host–guest complexes with noble metal halides such as AuBr_4_^−^, AuCl_4_^−^, and PdCl_4_^2−^.^[7]^ Guo et al. extracted gold selectively based on photoreduction methods using carbon nitride.^[8]^ Various kinds of metal–organic frameworks (MOFs) and covalent organic frameworks (COFs) were also exploited in gold capture from aqueous solutions.[^9^, ^10^, ^11^] The simple pyridine‐carboxylic acid, niacin was also used to precipitate gold halides selectively from mixed‐metal acidic solutions.^[12]^ Love and co‐workers developed a simple primary amide and showed that gold was recovered selectively from a mixture of metals representative of e‐waste by solvent extraction.^[13]^ Following on from this work, a method was developed for the selective precipitation of Au and Pt using a simple diamide.^[14]^ In this case, the direct and selective precipitation of metal complexes from acidic leach solutions was achieved and different NMs could be targeted according to the acid concentration.

While new processes that facilitate the separation of individual NMs from mixed‐metal leach solutions are being increasingly realized, environmentally benign methods for the leaching of NMs are less established. Industrial gold leaching still involves the extensive use of cyanide to form the soluble coordination complex NaAu(CN)_2_, and the leaching of gold from e‐waste requires strongly oxidizing acids such as aqua regia. However, new methods for the dissolution of NMs are being developed. For example, the dissolution of silver using glucose solution has been studied by Pradeep and co‐workers, although in this process, only 0.7 ppm of silver was leached using a 27.8 mm glucose solution after 7 days.^[15]^ Binnemans and co‐workers used highly concentrated acidic solutions of aluminum chlorides and nitrates for the dissolution of gold and platinum group metals.^[16]^ Lin et al. reported an “organic aqua regia” method comprising pyridine, dimethylformamide, or imidazole solutions of thionyl chloride (SOCl_2_), which showed high selectivity towards Pt/Au/Pd and Au/Pd mixtures.^[17]^ Serpe et al. exploited iodine in the presence of sulfur‐donor ligands such as dithioxamides or tetraalkylthiuram disulfides to oxidize Au^0^ to Au^III^ and effect its dissolution.^[18]^ Repo and co‐workers developed the use of pyridine thiols and H_2_O_2_ in ethanol solutions to dissolve gold.^[19]^ Recently, this same group achieved the dissolution of gold in ethanol using 2‐mercaptobenzimidazole as a ligand in the presence of catalytic quantities of iodine, taking around 13 h to dissolve gold quantitatively.^[20]^ Yang and co‐workers exploited *N*‐bromosuccinimide as an oxidant in pyridine to dissolve gold in water at pH 8.^[2]^ Recently, Chen et al. described the dissolution of NMs under photocatalytic conditions using TiO_2_ and ZnO photocatalysts.^[21]^ In this latter case, highly oxidizing organic radicals derived from acetonitrile and dichloromethane were formed by photocatalysis that ultimately resulted in the dissolution of NMs as their chloridometalates; unfortunately, the destruction of the organic solvents is a requirement for this dissolution method.

Herein, we report the very rapid dissolution of elemental Au, Pd, and Cu as their chloridometalates in various organic solvents using a mixture of triphenyldichlorophosphorane (Ph_3_PCl_2_) and hydrogen peroxide. Similarly, rapid dissolution of Au and Cu is seen using mixtures of another chlorinating agent, oxalyl chloride, and H_2_O_2_ in organic solvents. Regeneration of the spent triphenyldichlorophosphorane (as phosphine oxide) is achieved using oxalyl chloride. The selective transport and precipitation of NMs were achieved using biphasic mixtures, allowing for the facile separation of Au, Pt, and Pd.

## Results and Discussion

### Metal dissolution reactions with Ph_3_PCl_2_/H_2_O_2_

**Figure 1:**
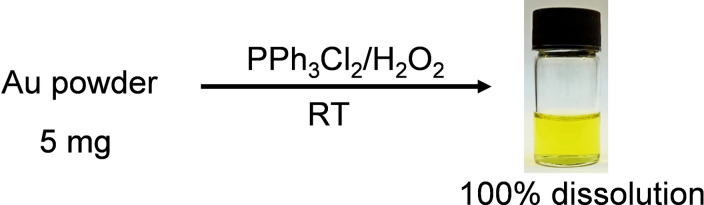
Dissolution of 5 mg of Au powder in 3 mL acetonitrile in 3 min at room temperature (RT) using 150 mg of Ph_3_PCl_2_ and 50 μL of H_2_O_2_.

The investigation was initiated with Au dissolution experiments. The reaction between fine Au powder (0.025 mmol) and Ph_3_PCl_2_ (0.450 mmol) in acetonitrile at room temperature (RT) results in 25 % gold dissolution over 24 h (Table 1, entry 1). However, the addition of H_2_O_2_ (50 μL, 32 wt %) to this reaction mixture causes a significant acceleration of gold dissolution; in this latter case, 100 % dissolution is achieved in just 3 min at RT (Table 1, entry 2 and Figure 1). The amount of Ph_3_PCl_2_ was varied and while 100 % dissolution of gold still occurs, the time taken for this process increases with decreasing concentrations of Ph_3_PCl_2_ (Table 1, entries 2–5). A variety of reactions were undertaken using different solvents (Table 1, entries 6–11). Excellent dissolution of gold is seen using polar aprotic solvents such as CH_3_CN, CH_2_Cl_2_, and tetrahydrofuran (THF), whereas a much slower dissolution rate is seen using 2‐methyltetrahydrofuran (Me‐THF); polar protic solvents resulted in either a slow dissolution rate (1‐octanol) or poor yields (ethanol). A time‐dependent study of Au leaching was undertaken using Ph_3_PCl_2_ (0.450 mmol) and H_2_O_2_ (50 μL) in acetonitrile (Figure S1) and shows 55 % dissolution after 30 s. This overall dissolution rate (100 mg h^−1^) is significantly faster than the previous examples using 2‐mercaptobenzimidazole as a ligand in the presence of catalytic quantities of iodine (0.15 mg h^−1^)^[20]^ or with pyridine thiols and H_2_O_2_ (12.9 mg h^−1^).^[19]^

**Table 1:** Optimization of the dissolution of gold in organic solvents by mixtures of Ph_3_PCl_2_ and H_2_O_2_.^[a]^

Entry	Solvent	H_2_O_2_ [μL]	Ph_3_PCl_2_ [mmol]	*t* [min]	Dissolution yield [%]
**1**	acetonitrile	0	0.450	1440	25
**2**	acetonitrile	50	0.450	3	100
**3**	acetonitrile	50	0.300	20	100
**4**	acetonitrile	50	0.150	40	100
**5**	acetonitrile	50	0.075	150	100
**6**	chloroform	50	0.450	3	92
**7**	dichloromethane	50	0.450	3	100
**8**	ethanol	50	0.450	1440	36
**9**	THF	50	0.450	5	100
**10**	Me‐THF	50	0.450	480	100
**11**	1‐octanol	50	0.450	180	100

[a] Reaction conditions: gold 5 mg, 900 rpm, 32 wt % H_2_O_2_, RT, 3 mL solvent.

**Figure 2:**
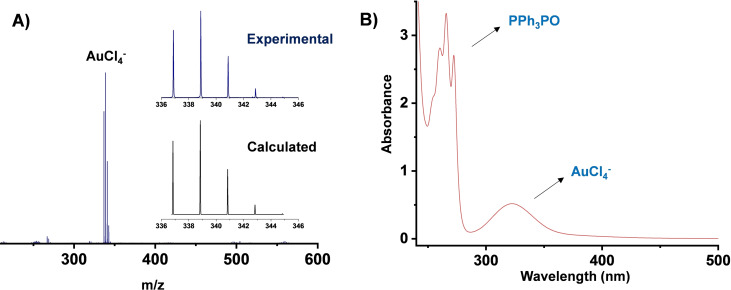
(A) Negative ion ESI‐MS of the acetonitrile solution after gold dissolution using Ph_3_PCl_2_/H_2_O_2_ with observed and calculated isotopic distribution patterns for AuCl_4_^−^. (B) UV/Vis spectrum of the acetonitrile solution after gold dissolution using Ph_3_PCl_2_/H_2_O_2_ showing the presence of AuCl_4_^−^ and Ph_3_PO.

To understand which gold complexes are formed during the dissolution process, electro‐spray ionization mass spectrometry (ESI‐MS) of the acetonitrile solution after Au dissolution by Ph_3_PCl_2_/H_2_O_2_ was recorded. In the negative ion mode, the sole formation of AuCl_4_^−^ is observed (Figure 2A), whereas the positive ion mode reveals the presence of (PPh_3_O)_2_H^+^ and (PPh_3_O)H^+^ (Figure S2A). This speciation in solution is supported by the UV/Vis spectrum of the same solution, in which the presence of AuCl_4_^−^ is confirmed by a peak at 323 nm (Figure 2B, and Figure S2B for comparison with standard AuCl_4_^−^) along with absorption due to Ph_3_PO in the range of 260–280 nm. This is further evidenced by a ^31^P{^1^H} nuclear magnetic resonance (NMR) spectrum that shows a resonance at 40.1 ppm (Figure S3) attributed to the formation of (PPh_3_O)_2_H^+^.^[22]^ It is therefore evident that the dissolution conditions oxidize Au^0^ to form the Au^III^ complex AuCl_4_^−^. An overall reaction equation is presented in Figure S4.

The dissolution of Pd (on carbon, 10 wt %) was studied using the same protocol and complete dissolution is observed after 3 min in either acetonitrile or dichloromethane (Figure S5 and Table S1). A time‐dependent dissolution study was performed using Ph_3_PCl_2_ (0.450 mmol) and H_2_O_2_ (50 μL) in acetonitrile (Figure S6) and shows around 68 % dissolution after 30 s, which is faster than Au dissolution (55 % after 30 s) under similar conditions; however, the time taken for complete dissolution generates a rate of 100 mg h^−1^, which is similar to that for Au dissolution. The dissolution rate of Pt (on carbon, 5 wt %) is slower, taking 6 h to dissolve 100 % of the Pt in acetonitrile in the absence of H_2_O_2_ (Table S2, Figures S7 and S8 A). The inclusion of H_2_O_2_ into the leachate decreases the rate of dissolution, with only 87 % of Pt dissolved after 24 h (Table S2 and Figure S8B). This may be due to the peroxide being oxidized by Pt^IV^ halides, thereby limiting overall dissolution. This observation may deliver an unexpected advantage by presenting a route for the kinetic separation of Pd and Pt.

To characterize the complexes formed, ESI‐MS for the Pd and Pt leached solutions were studied in the negative mode in acetonitrile from which a range of chloridometalate ions are detected (Figures S9 and S10). Evaporation of these leach solutions, dissolution in chloroform, and contacting with 6 m HCl results in the phase‐transport of Pd [99.5 % by inductively coupled plasma optical emission spectroscopy (ICP‐OES)] and Pt (99.2 % by ICP‐OES) into the aqueous phase (Figure S11) and the retention of Ph_3_PO in the organic phase. The UV/Vis spectra of these complexes are similar to those of PdCl_4_^2−^ and PtCl_6_^2−^ in 6 m HCl (Figures S12 and S13), supporting the formation of Pd^II^ and Pt^IV^ chloridometalates. The ^31^P{^1^H} NMR spectrum of the organic layer shows that Ph_3_PO remains in the organic layer (Figure S14). The formation of Pd and Pt chloridometalates and their phase transport into 6 m HCl allows their separation to be undertaken using our previously described precipitation method.^[14]^ As such, treating the Pt‐containing acidic aqueous phase with the diamide PhC(O)NMe(CH_2_CH_2_)NMeC(O)Ph (L) results in the complete precipitation of [HL]_2_PtCl_6_ (Figure S15). It is therefore evident that the combination of this new leaching protocol with our previously reported selective precipitation method could result in a more sustainable and selective recovery of NMs.

Lastly, the leaching of elemental copper was also undertaken using the same protocol (Ph_3_PCl_2_/H_2_O_2_/acetonitrile) which results in its complete dissolution after 2 min at RT (Figure S16). As with the above, the organic leach solution was contacted with 2 m HCl to transport the metal complex into the aqueous phase. The UV/Vis spectrum of this aqueous phase is analogous to that of CuCl_4_^2−^ in 2 m HCl (Figure S17).

### Application to e–waste and nanoparticle dissolution

**Figure 3:**
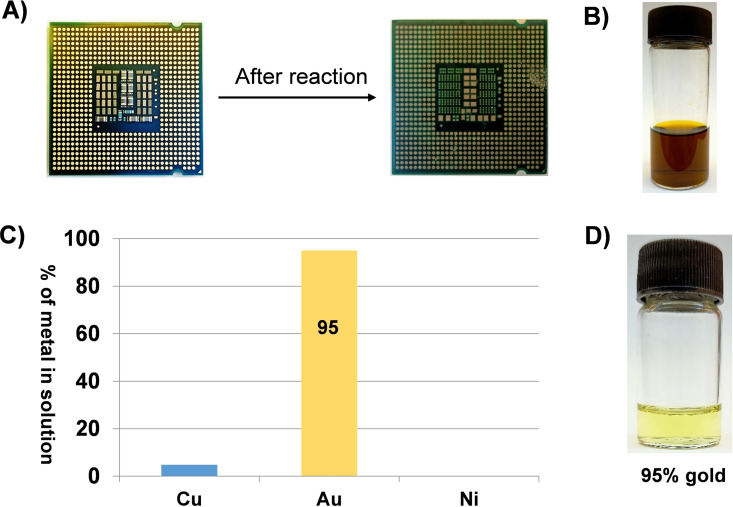
(A) CPU before and after the reaction. (B) E‐waste leachate solution from the CPU. (C) ICP‐OES data of the separated Au solution in the organic layer. (D) The 95 % Au solution after separation.

As the dissolution of e‐waste would result in mixtures of metals such as Cu and Au, the phase separation of these metals was studied after their leaching into acetonitrile. The reaction of a 1 : 4 Au/Cu (5 mg:20 mg) mixture with Ph_3_PCl_2_/H_2_O_2_ in acetonitrile results in the complete dissolution of the metals (Figure S18A). Subsequent addition of dichloromethane and 2 m HCl causes a distinct phase separation in which [(Ph_3_PO)_2_H][AuCl_4_] (99.8 % by ICP‐OES, Figure S18B) remains in the organic phase while CuCl_4_^2−^ is transported into the aqueous phase (Figure S18A). Given the straightforward nature of this model Cu/Au separation, the leaching and separation protocol was applied to e‐waste central processing units (CPU). Soaking a CPU in an acetonitrile solution of Ph_3_PCl_2_/H_2_O_2_ results in metal dissolution from the surface (Figure 3A) and the formation of a leachate (Figure 3B) comprising Cu (4017 ppm), Ni (720 ppm), and Au (74 ppm). Dichloromethane and 2 m HCl were added to the acetonitrile leach solution and, after separating the phases and washing the organic phase with 2 m HCl, an organic solution comprising 95 % pure gold is obtained.

The increased growth in high‐efficiency compact storage devices, medical diagnostics, photovoltaics, imaging, and other gold‐based nanotechnology[^23^, ^24^, ^25^] is leading to increased generation of waste nanoparticulate gold. As such, a precipitate of citrate‐reduced AuNPs was obtained using NaCl‐induced aggregation to make a simulated nano‐waste for leaching by the above protocol.^[25]^ It was found that a mixture of Ph_3_PCl_2_ and H_2_O_2_ in acetonitrile at RT rapidly and completely leaches the simulated nano‐waste (Figure S19), thus demonstrating that this procedure can be exploited to replace classical leaching methods for this growing waste stream.

### Recycling of reagents using oxalyl chloride

After the dissolution of Au and its separation from other metals, it is necessary to reduce the [(Ph_3_PO)_2_H][AuCl_4_] produced to isolate metallic gold and to regenerate the triphenyldichlorophosphorane oxidant Ph_3_PCl_2_ from the oxide Ph_3_PO. In the first instance, NaBH_4_ was added to the Au‐containing leach solution which results in the rapid reduction and precipitation of metallic gold (99 %) (Figure S20A). Triphenylphosphine oxide remains in the organic solution after reduction, as confirmed by a resonance at 27.5 ppm in the ^31^P{^1^H} NMR spectrum (Figure S20B).

**Figure 4:**
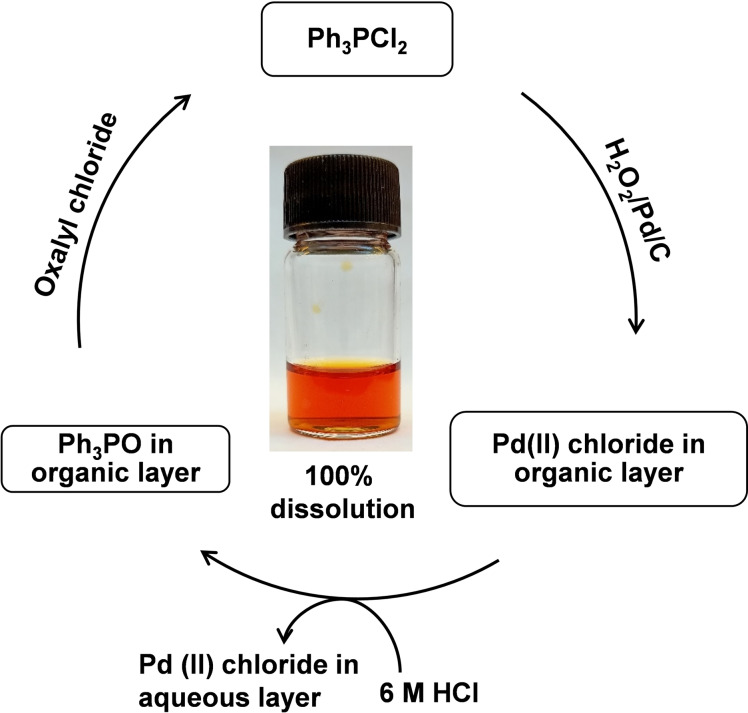
Schematic representation of dissolution of Pd using Ph_3_PO and oxalyl chloride and phase‐transfer using 6 m HCl.

The recycling of the oxidant Ph_3_PCl_2_ is necessary to ensure the atom‐economy and circularity of this method. In theory, chlorine gas could be used to convert the phosphine oxide Ph_3_PO formed during the leaching reaction into the chlorophosphorane^[26]^ but, due to the difficulty in handling this toxic gas, oxalyl chloride was used as a surrogate.[^27^, ^28^] To exemplify this, Ph_3_PCl_2_ was prepared by reacting oxalyl chloride (0.45 mmol) with Ph_3_PO (0.90 mmol) in acetonitrile, after which the solution was purged with nitrogen gas to remove any carbon monoxide released by oxalyl chloride decomposition. The addition of 50 mg of Pd/C and 50 μL H_2_O_2_ to this reaction mixture (Figure 4) results in rapid and quantitative Pd dissolution. This cycle was repeated twice with complete Pd dissolution seen in both cases (Figure S21). Similarly, quantitative dissolution cycles are also seen for Au (Figure S22A,B). Rapid dissolution of gold is also observed using oxalyl chloride (0.45 mmol) and H_2_O_2_ (50 μL) in organic solvents in the absence of Ph_3_PCl_2_ (Table S3), with 100 % dissolution of gold occurring in 3 min at RT. A time‐dependent dissolution study (Figure S23) revealed around 58 % dissolution is achieved after 30 s, which is similar to that seen using PPh_3_Cl_2_/H_2_O_2_ mixtures (55 %). Formation of HAuCl_4_ is confirmed by analysis of the ESI‐MS and UV/Vis spectra of the leached solution in acetonitrile (Figure S24A,B). Formation of HAuCl_4_ is again supported by UV/Vis spectra after evaporating the acetonitrile leach solution and redissolving in 2 m HCl (Figure S25A). The quantitative precipitation of HAuCl_4_ was also achieved using PhC(O)NMe(CH_2_CH_2_)NMeC(O)Ph (L) (Figure S25B).^[14]^ A schematic representation of the selective separation of gold using PPh_3_Cl_2_ and H_2_O_2_ (method I) and oxalyl chloride and H_2_O_2_ (method II) is shown in Scheme S1.

Oxalyl chloride is also found to leach Pd from carbon over 24 h, but in this case, the resulting mixture is reduced to Pd black due to the presence of CO (Figure S26).[^29^, ^30^] The dissolution of Pt on carbon using oxalyl chloride is slower than with Ph_3_PCl_2_, taking 6 h to dissolve 88 % of the Pt in acetonitrile (Figure S27A). As with Ph_3_PCl_2_, the addition of H_2_O_2_ hinders dissolution rates, with only 83 % of Pt leached after 24 h (Figure S27B). Formation of the Pt^IV^ chloride complex is confirmed by UV/Vis spectra after evaporating the leach solution and redissolving in 6 m HCl (Figure S28). Lastly, copper (5 mg) is also dissolved rapidly (2 min) using oxalyl chloride (0.45 mmol) and H_2_O_2_ (50 μL) in acetonitrile (Table S4). Formation of a Cu^II^ chloride complex was confirmed by UV/Vis spectra after evaporating the leach solution and redissolving in 2 m HCl (Figure S29).

## Conclusion

We have demonstrated new methods for the dissolution of Au, Pd, Pt, and Cu using mixtures of Ph_3_PCl_2_/H_2_O_2_ or (COCl)_2_/H_2_O_2_ in organic solvents such as acetonitrile. Both processes dissolve noble metals (NMs) quantitatively to produce organic‐soluble chloridometalate complexes and are extremely rapid (except for Pt), with complete dissolution of the metals occurring in minutes. Significantly, simple phase‐transfer operations allow for the separation of Au from other metals. The regeneration of the triphenyldichlorophosphorane and the recycling of the organic solvent results only in the consumption of the relatively inexpensive hydrogen peroxide and oxalyl chloride, although the latter could, in principle, be replaced by chlorine gas. Similar dissolution of other platinum group metals such as Ir and Rh is likely using this procedure. These findings suggest that this leaching protocol has the potential to be exploited in the sustainable purification of NMs from ores, spent catalysts, and electronic and nano‐wastes.

## Experimental Section

### Typical dissolution procedure

In a typical dissolution process, 5 mg of gold (0.025 mmol) was added to a mixture of Ph_3_PCl_2_ (150 mg, 0.45 mmol) and H_2_O_2_ (50 μL, 0.15 mmol) in acetonitrile (3 mL) at room temperature while stirring (900 rpm). Pd and Cu were treated by the same protocol. Oxalyl chloride (0.45 mmol) could be used in place of Ph_3_PCl_2_ using the same protocol. Pt was dissolved using two protocols: (a) 100 mg of fine Pt/C powder (5 wt % Pt) (5 mg, 0.025 mmol), Ph_3_PCl_2_ (150 mg, 0.45 mmol), and (b) 100 mg of fine Pt/C powder (5 wt % Pt) (5 mg, 0.025 mmol), Ph_3_PCl_2_ (150 mg, 0.45 mmol) and H_2_O_2_ (50 μL, 0.15 mmol) in acetonitrile (3 mL). Note that any H_2_O_2_ remaining on completion of the dissolution experiments was destroyed prior to disposal of the mixtures.

### Dissolution of CPU

The metals present in the CPU were dissolved using a modification of the typical procedure described above. The CPU was soaked in a mixture of Ph_3_PCl_2_ (150 mg, 0.45 mmol) and H_2_O_2_ (50 μL, 0.15 mmol) in acetonitrile (6 mL) for 4 h at room temperature. After this period, the solution was decanted and the CPU was soaked for a second time for 4 h in a fresh Ph_3_PCl_2_/H_2_O_2_/acetonitrile solution. The leach solutions were combined and analyzed by ICP‐OES to determine the extent of metal dissolution from the CPU.

## Conflict of interest

The authors declare no conflicts of interest.

1

## Supporting information

As a service to our authors and readers, this journal provides supporting information supplied by the authors. Such materials are peer reviewed and may be re‐organized for online delivery, but are not copy‐edited or typeset. Technical support issues arising from supporting information (other than missing files) should be addressed to the authors.

Supporting Information

## Data Availability

The data that support the findings of this study are available in the supplementary material of this article.
