# Supplementary material for: Rapid Dissolution of Noble Metals in Organic Solvents
Source: ChemSusChem. 2022 Sep 1;15(20):e202201285. doi: 10.1002/cssc.202201285 (PMC9804267; doi:10.1002/cssc.202201285)
Supplement: Supplementary file 1 — Supporting Information [file CSSC-15-0-s001.pdf]

ChemSusChem

Supporting Information

Rapid Dissolution of Noble Metals in Organic Solvents

Abhijit Nag, Carole A. Morrison, and Jason B. Love*© 2022 The Authors. ChemSusChem published by Wiley-VCH GmbH. This is an open access article under the terms of the Creative Commons Attribution License, which permits use, distribution and reproduction in any medium, provided the original work is properly cited.

Materials and Methods

All reagents and solvents were used as obtained from Sigma-Aldrich, Fisher Scientific UK, Alfa Aesar, Acros Organics or VWR International. Deionised water was taken from a MilliQ purification system.

Calculation of Leaching Yield. Au (5 mg), Pd (50 mg Pd/C), Pt (100 mg Pt/C) and Cu (5 mg) were leached completely using aqua regia, with the yield of dissolution calculated according to the equation below:

$$\% \text{ of Yield} = \frac{\text{The amount of NMs leaching by the current study}}{\text{The amount of NMs leaching by the current study aqua regia}} \times 100$$

Instrumentation

ICP-OES. Quantitative metal analysis was carried out on a Perkin Elmer Optima 5300DC Inductively Coupled Plasma Optical Emission Spectrometer. For the measurements of metals in organic solvents 1-methoxy-2-propanol was used; for the aqueous measurements 2% HNO_3 was used. For the organic sample measurements, the argon plasma conditions were 1550 W RF power, with gas flows of 17, 1.0, and 0.5 L min^{-1} for plasma, auxiliary, and nebuliser flow, respectively. For the aqueous sample measurements, the argon plasma conditions were 1550 W RF power, with gas flows of 15, 1.07, and 0.9 L min^{-1} for plasma, auxiliary, and nebuliser flows, respectively. Prior to sample data collection, the instrument was calibrated (R^2=0.9999) with metal standards, obtained from VWR International, Merck Millipore, or Sigma-Aldrich.

ESI-MS. ESI-MS measurements were recorded in positive and negative-ion mode using a Bruker ESI Micro-TOF spectrometer equipped with Liquid Chromatography (LC). All mass spectra were analyzed using Data Analysis software version 4.4 (Bruker Daltonics) with the ion peaks assigned manually.

UV-visible Spectrophotometry. The UV-vis experiments were analysed against a solvent blank over the range 200-1100 nm on a Shimadzu UV-1900 spectrometer.

NMR Spectroscopy. $^{31}P\{^{1}H\}$ NMR spectra were recorded on a Bruker Pro 500 spectrometer operating at 202 MHz at 300 K in CD_3CN. 85 % H_3PO_4 was used as an external standard: 0.00 ppm.

Figures and Tables.

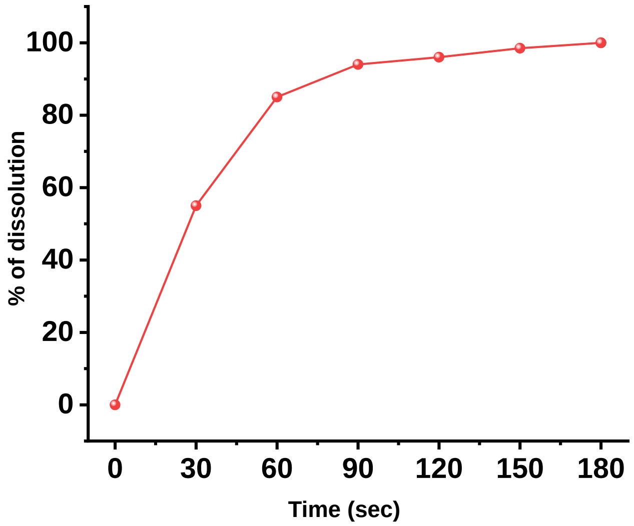

Figure S1. Time-dependent dissolution of Au for the reaction mixture comprising fine Au powder (5 mg, 0.025 mmol), Ph_3PCl_2 (150 mg, 0.45 mmol) and H_2O_2 (50 µL, 0.15 mmol) in 3 mL acetonitrile at RT.

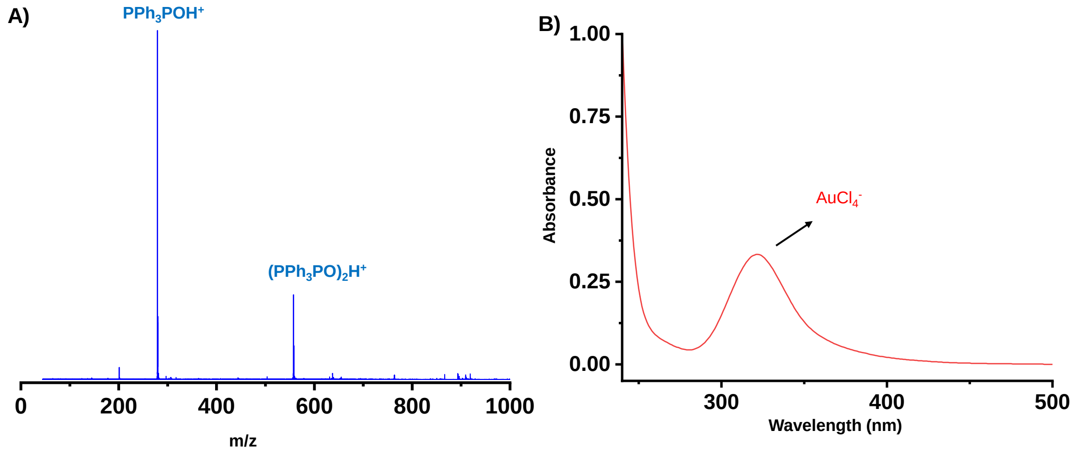

Figure S2. A) Positive-ion ESI-MS of the Au leach solution showing the presence $(Ph_3PO)_2H^+$ and Ph_3POH^+. **B)** UV-vis spectrum of a standard solution of $HAuCl_4$ in acetonitrile.

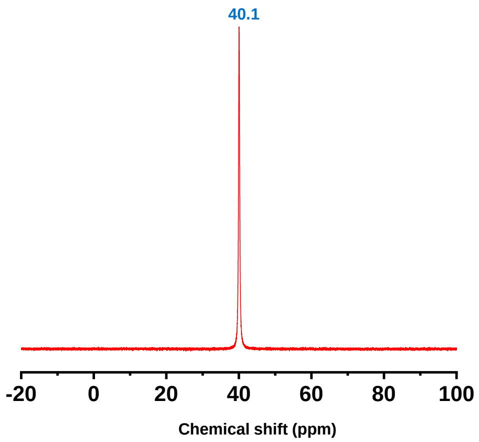

Figure S3. $^{31}P\{^{1}H\}$ NMR spectrum of the Au leach solution after reaction. The peak at 40.1 ppm is assigned to the cation $(Ph_3PO)_2H^+$.

$$4Ph_3PCl_2 + 3H_2O_2 + 2Au = 2[(Ph_3PO)_2H][AuCl_4] + 2H_2O$$

Figure S4. An overall reaction equation of the Au dissolution process. The structure of $[(Ph_3PO)_2H][AuCl_4]$ was reported by Dutton et al.[1]

Entry	Solvent	H_2O_2 (µL)	Ph_3PCl_2 (mmol)	Time (min)	Dissolution Yield (%)
**1**	Acetonitrile	50	0.450	3	100
**2**	Dichloromethane	50	0.450	3	100

Table S1: The dissolution of Pd in organic solvents by mixtures of Ph_3PCl_2 and H_2O_2.

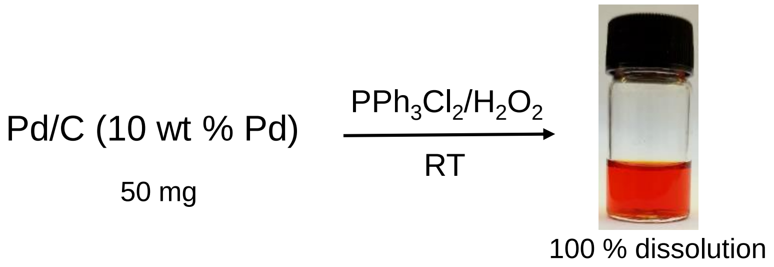

Figure S5: Dissolution of Pd from a mixture comprising Pd/C powder (50 mg, 10 wt% Pd), Ph_3PCl_2 (150 mg, 0.45 mmol) and H_2O_2 (50 µL, 0.15 mmol) for 3 minutes at RT in 3 mL acetonitrile.

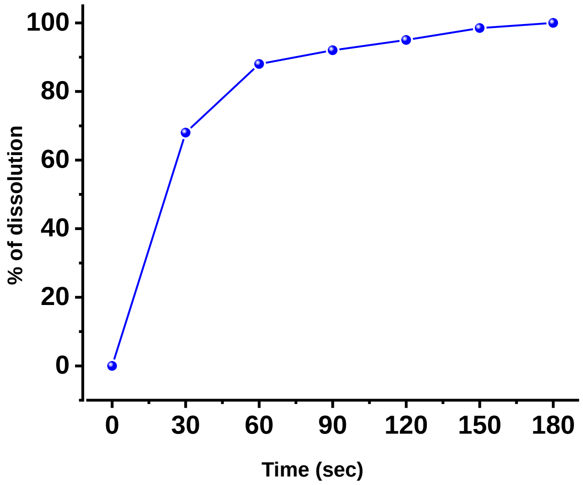

Figure S6: Time-dependent dissolution of Pd for the reaction mixture comprising 50 mg of fine Pd/C powder (10 wt% Pd, 5mg, 0.047 mmol), Ph_3PCl_2 (150 mg, 0.45 mmol) and H_2O_2 (50 µL, 0.15 mmol) in 3 mL acetonitrile at RT.

Entry	Solvent	H_2O_2 (µL)	Ph_3PCl_2 (mmol)	Time (hrs)	Dissolution Yield (%)
**1**	Acetonitrile	0	0.450	6	100
**2**	Acetonitrile	50	0.450	24	87

Table S2: The dissolution of Pt in organic solvents

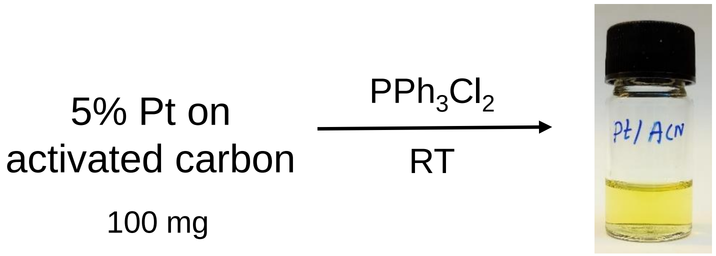

Figure S7. Dissolution of Pt from a mixture comprising Pt/C powder (100 mg, 5 wt% Pt) powder, Ph_3PCl_2 (150 mg, 0.45 mmol) and H_2O_2 (50 µL, 0.15 mmol) for 3 minutes at RT in 3 mL acetonitrile.

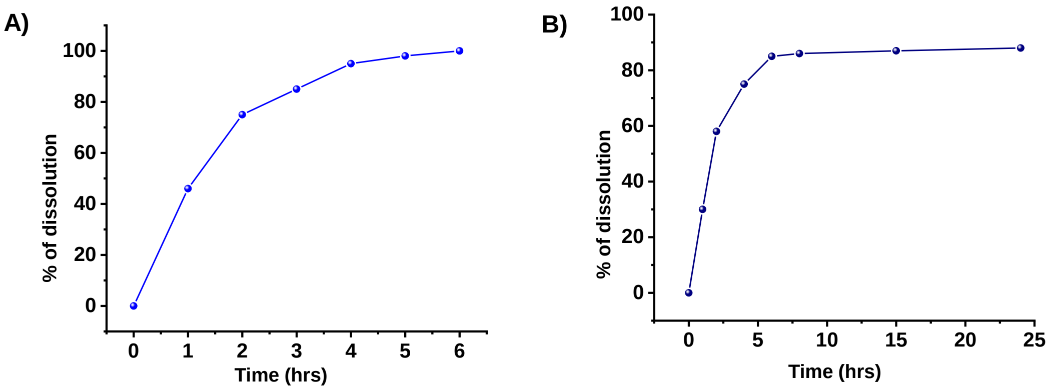

Figure S8. Time-dependent dissolution of Pt for the reaction mixture comprising **A)** 100 mg of fine Pt/C powder (5 wt% Pt) (5 mg, 0.025 mmol), Ph_3PCl_2 (150 mg, 0.45 mmol) and **B)** 100 mg of fine Pt/C powder (5 wt% Pt) (5 mg, 0.025 mmol), Ph_3PCl_2 (150 mg, 0.45 mmol) and H_2O_2 (50 µL, 0.15 mmol) in 3 mL acetonitrile at RT.

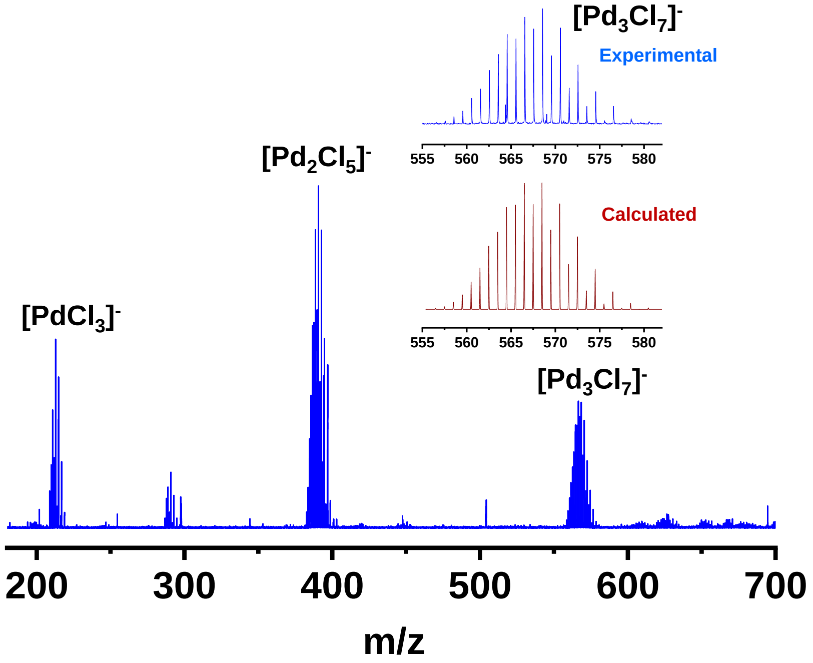

Figure S9. ESI MS of the Pd leached solution in negative mode in acetonitrile. Formation of $PdCl_3^-$, $Pd_2Cl_5^-$ and $Pd_3Cl_7^-$ were observed. Experimental and calculated isotopic pattern of $Pd_3Cl_7^-$ are provided in the inset of Figure S9.

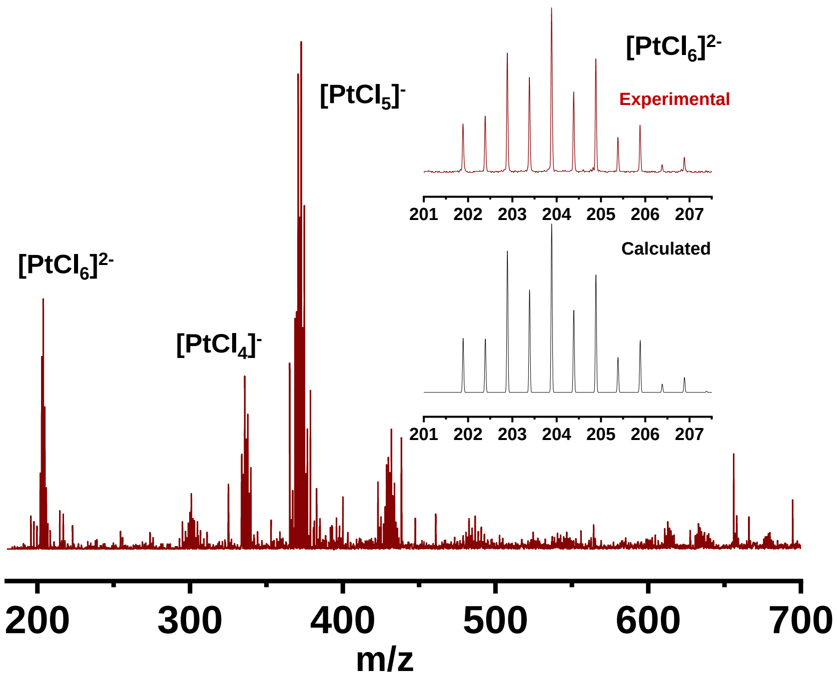

Figure S10. ESI MS of the Pt leached solution in negative mode in acetonitrile. Formation of $PtCl_6^{2-}$, $PtCl_5^-$ and $PtCl_4^-$ were observed. Experimental and calculated isotopic pattern of $PtCl_6^{2-}$ are provided in the inset of Figure S9.

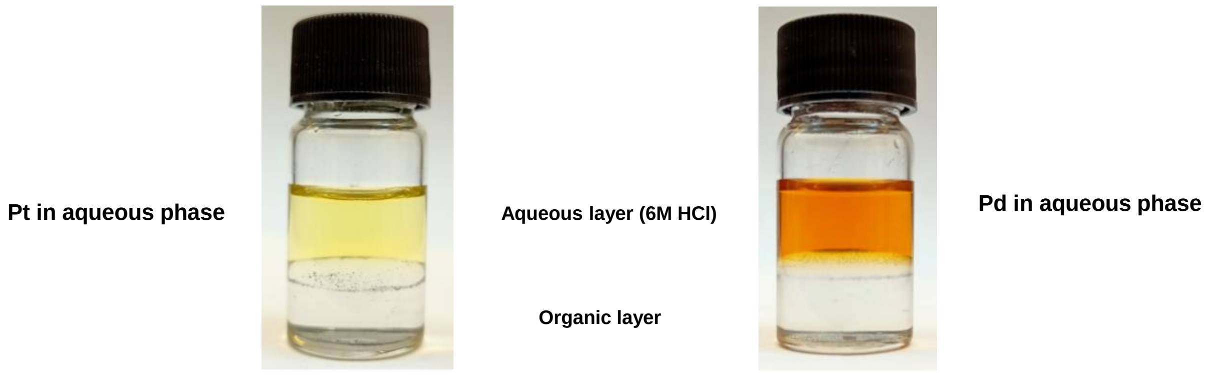

Figure S11. Phase separation of Pt (99.2%) and Pd (99.5%) chloridometalates from the organic phase ($CHCl_3$) into aqueous layer (6 M HCl). Note that the black particles are activated carbon from the original Pd/C and Pt/C samples.

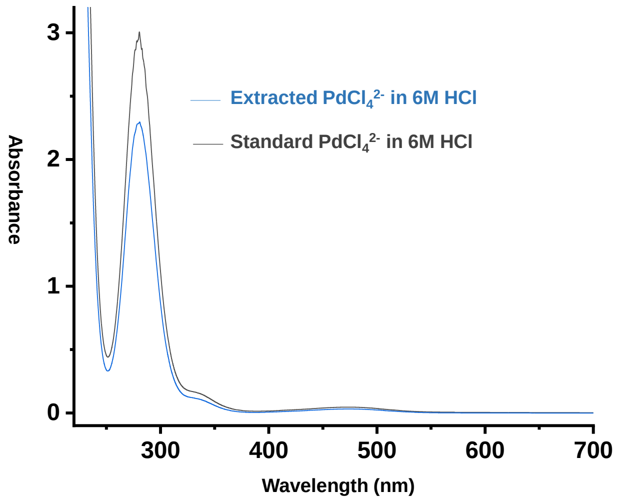

Figure S12. UV-vis spectra of standard $PdCl_4^{2-}$ in 6M HCl and its comparison with that arising from the leaching/extraction experiment.

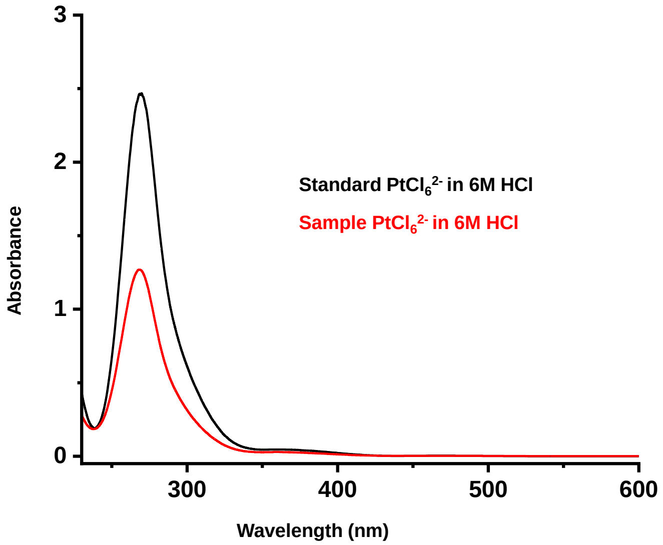

Figure S13. UV-vis spectra of standard Na_2PtCl_6 in 6M HCl and its comparison with that arising from the leaching/extraction experiment.

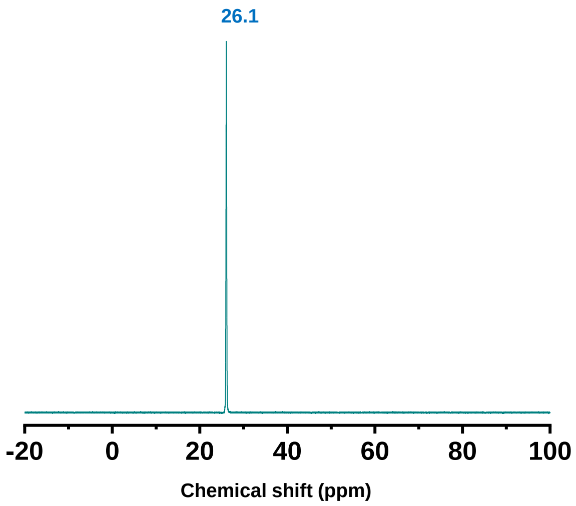

Figure S14. $^{31}P\{^1H\}$ NMR spectrum of triphenylphosphine oxide that remains in the organic layer after phase transfer of the chloridometalates.

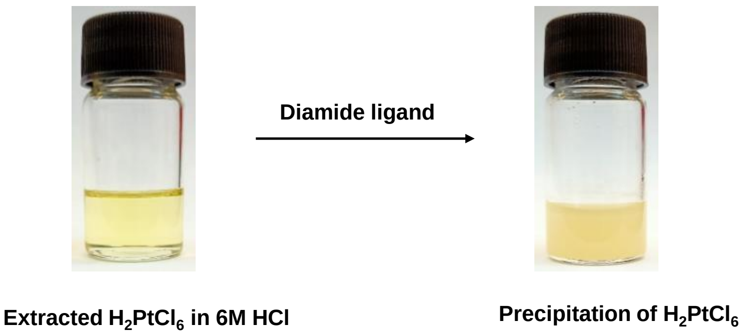

Figure S15. The use of the diamide $PhC(O)NMeCH_2CH_2NMeC(O)Ph$ (L) to precipitate Pt from the aqueous layer as $[HL]_2[PtCl_6]$.[2]

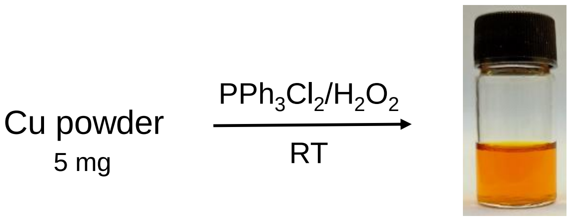

Figure S16. Dissolution of Cu from a mixture comprising Cu powder (5mg, 0.078 mmol), Ph_3PCl_2 (150 mg, 0.45 mmol) and H_2O_2 (50 µL, 0.15 mmol) for 2 minutes at RT in 3 mL acetonitrile.

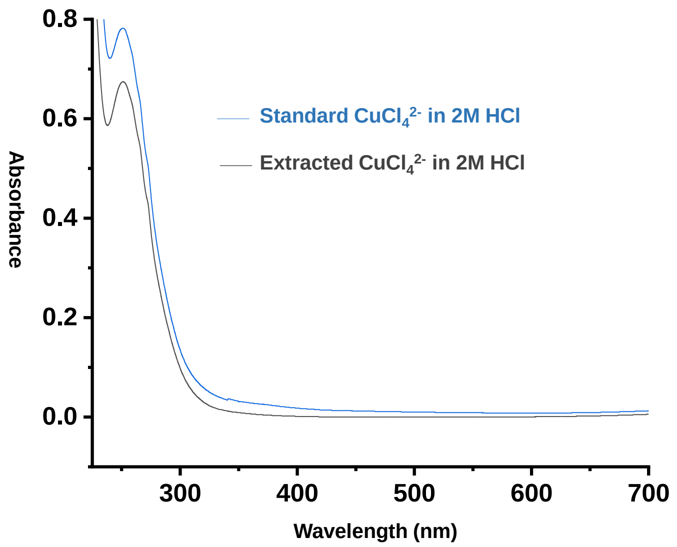

Figure S17. UV-vis spectra of standard $CuCl_4^{2-}$ in 2M HCl and its comparison with that arising from the leaching/extraction experiment.

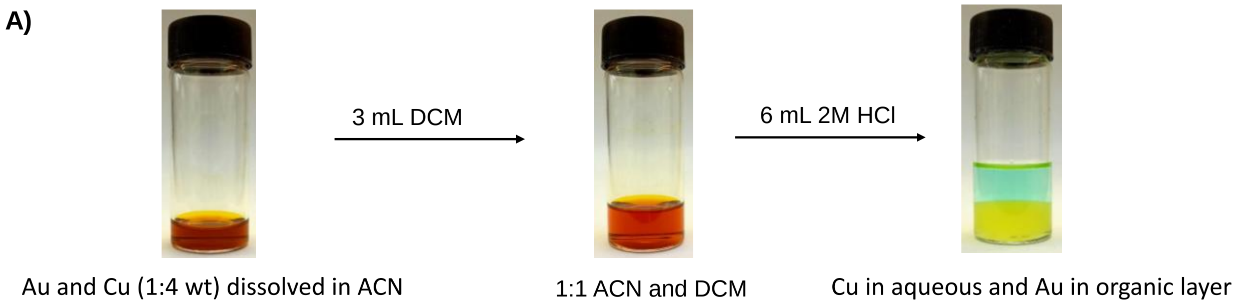

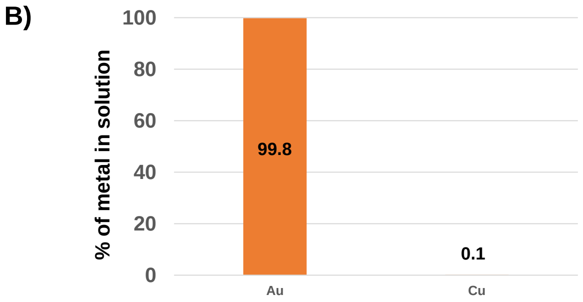

Figure S18. A) Separation of Au and Cu in aqueous and organic layer after leaching. **B)** Quantification of the percentage of metal that remains in the organic layer by ICP-OES.

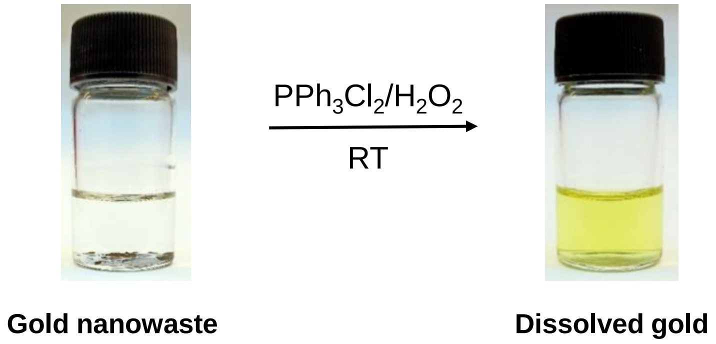

Figure S19. Dissolution of Au nanowaste using Ph_3PCl_2 (150 mg, 0.45 mmol) and H_2O_2 (50 µL, 0.15 mmol) for 3 minutes at RT in 3 mL acetonitrile.

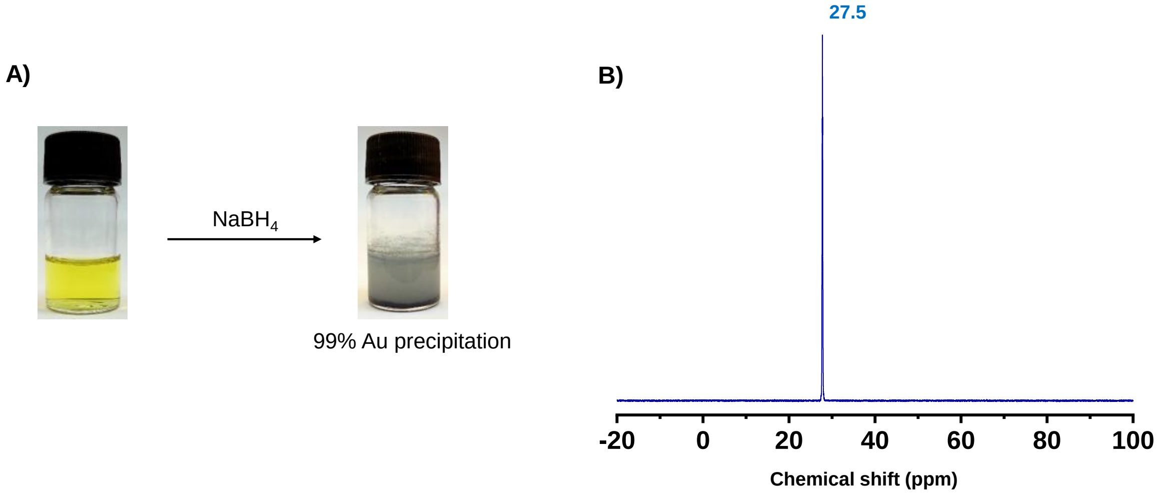

Figure S20. A) Reduction of $[(Ph_3PO)_2H][AuCl_4]$ using $NaBH_4$ (15 mg) in 3 mL acetonitrile to form a (black) Au precipitate (99%). **B)** $^{31}P\{^1H\}$ NMR spectrum of triphenylphosphine oxide that remains in acetonitrile after reduction.

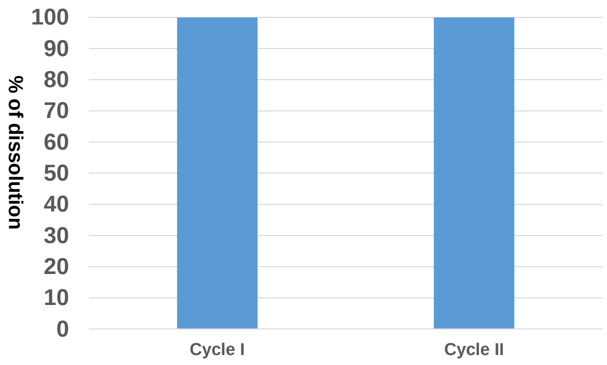

Figure S21. 100% dissolution of Pd in both cycle I and cycle II.

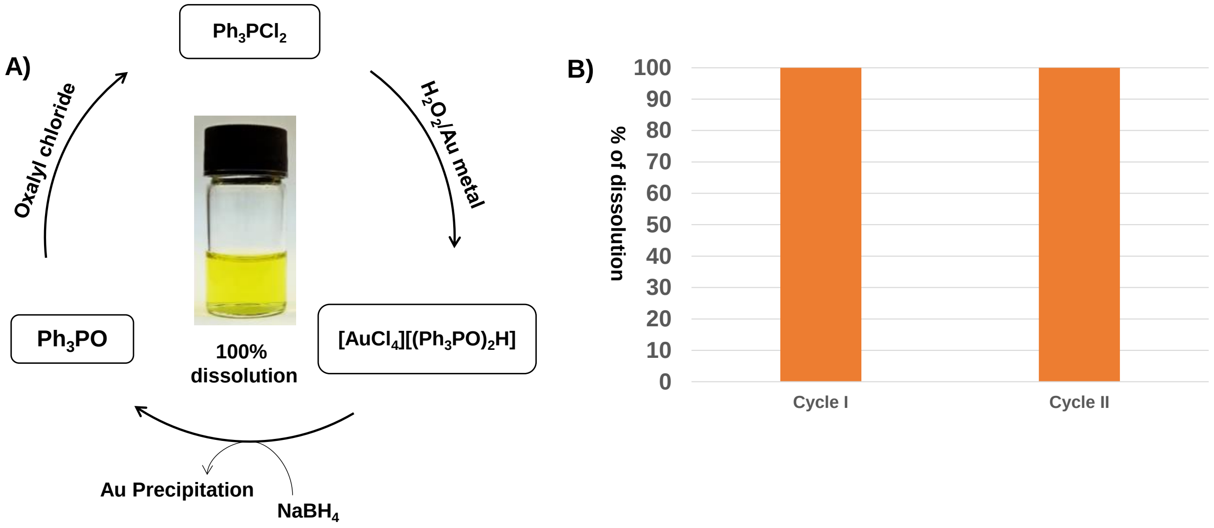

Figure S22. **A)** Schematic representation of dissolution of Au cycle using Ph_3PCl_2/H_2O_2 and the regeneration of Ph_3PCl_2 using oxalyl chloride. **B)** 100% dissolution of gold in both cycle I and cycle II.

Entry	Solvent	H_2O_2 (µL)	Oxalyl chloride (mmol)	Time (min)	Dissolution Yield (%)
**1**	Acetonitrile	50	0.450	3	100
**2**	Dichloromethane	50	0.450	5	100
**3**	Chloroform	50	0.450	5	100

Table S3: The dissolution of Au in organic solvents by mixtures of oxalyl chloride and H_2O_2.

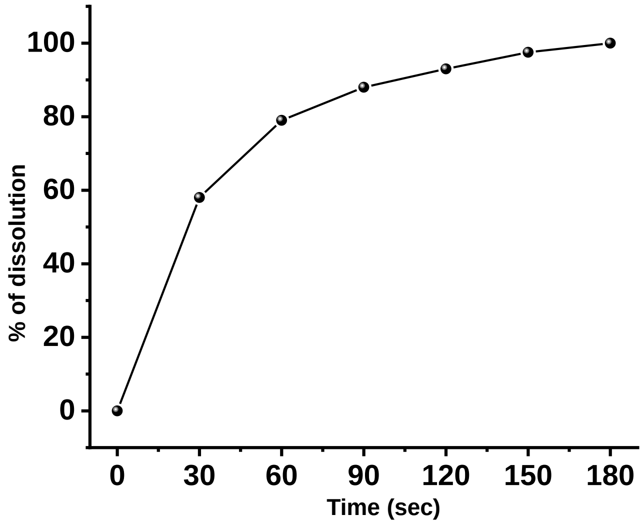

Figure S23. Time-dependent dissolution of Au for the reaction mixture comprising fine Au powder (5 mg, 0.025 mmol), oxalyl chloride (0.45 mmol) and H_2O_2 (50 µL, 0.15 mmol) in 3 mL acetonitrile at RT.

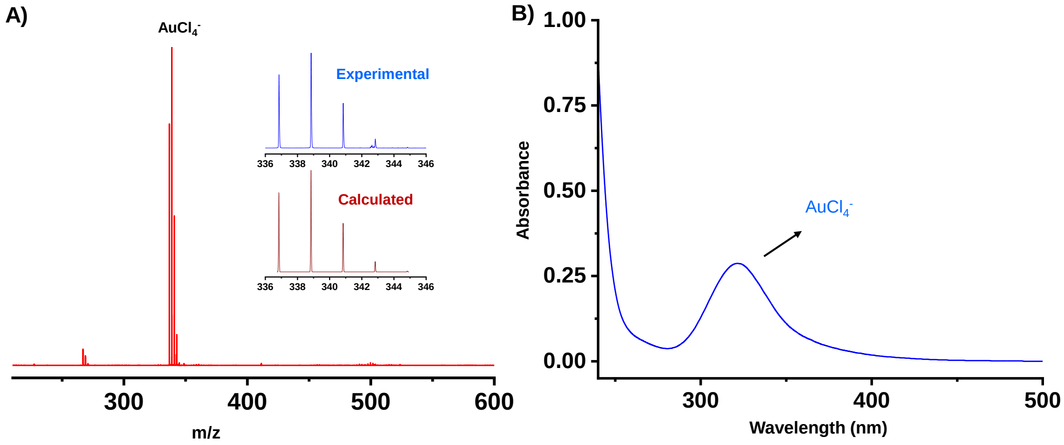

Figure S24. A) Negative ion ESI-MS of the acetonitrile solution after gold dissolution using $(COCl)_2/H_2O_2$ with observed and calculated isotopic distribution patterns for $AuCl_4^-$. B) UV-vis spectrum of the acetonitrile solution after gold dissolution using $(COCl)_2/H_2O_2$ showing the presence of $AuCl_4^-$.

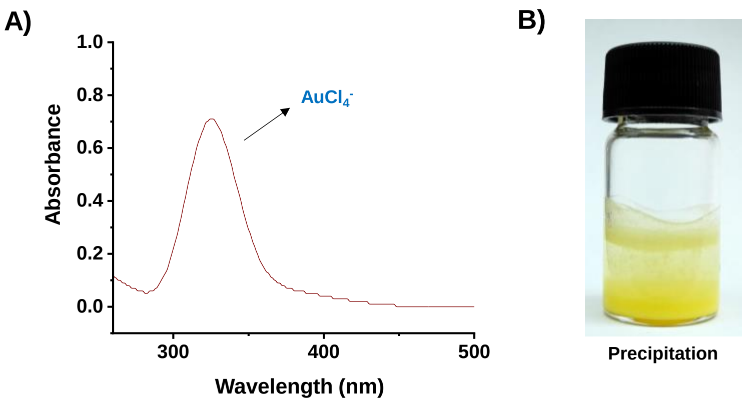

Figure S25. **A)** UV-vis spectra of $HAuCl_4$ after evaporating the leach solution (acetonitrile) and redissolving in 2M HCl. **B)** Precipitation of Au from 2M HCl using PhC(O)NMe(CH_2CH_2)NMeC(O)Ph (L) as [HL][$AuCl_4$].

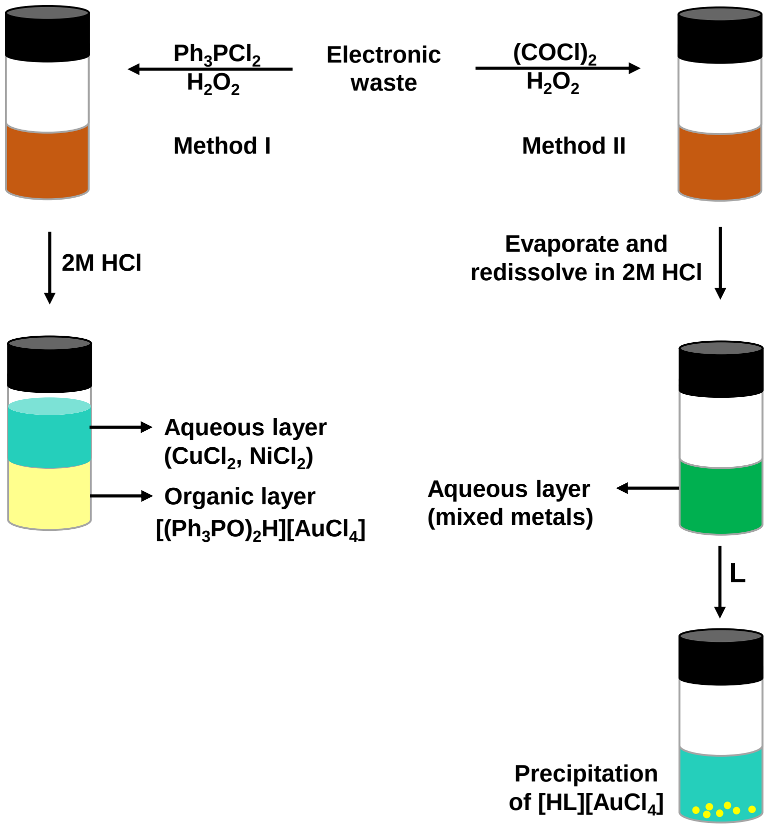

Scheme S1. Schematic representation of the selective gold recovery from electronic wastes using method I and method II.

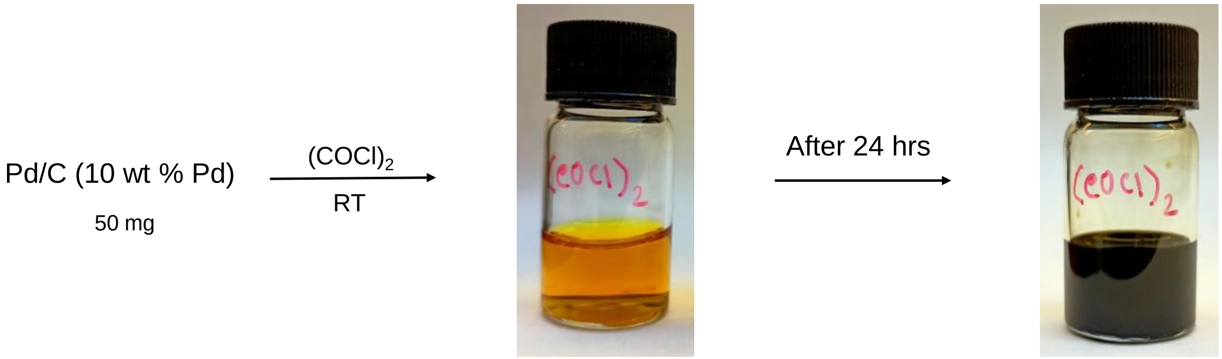

$PdCl_2 + CO + H_2O = 2HCl + Pd + CO_2$

Figure S26. Dissolution of Pd from a mixture comprising Pd/C powder (50 mg, 10 wt% Pd, 5mg, 0.047 mmol), and oxalyl chloride (0.45 mmol) after 24 hrs at RT in acetonitrile. The resulting solution was reduced to Pd black within 24 h. An overall reaction equation of the $PdCl_2$ reduction process is also provided.[3-4]

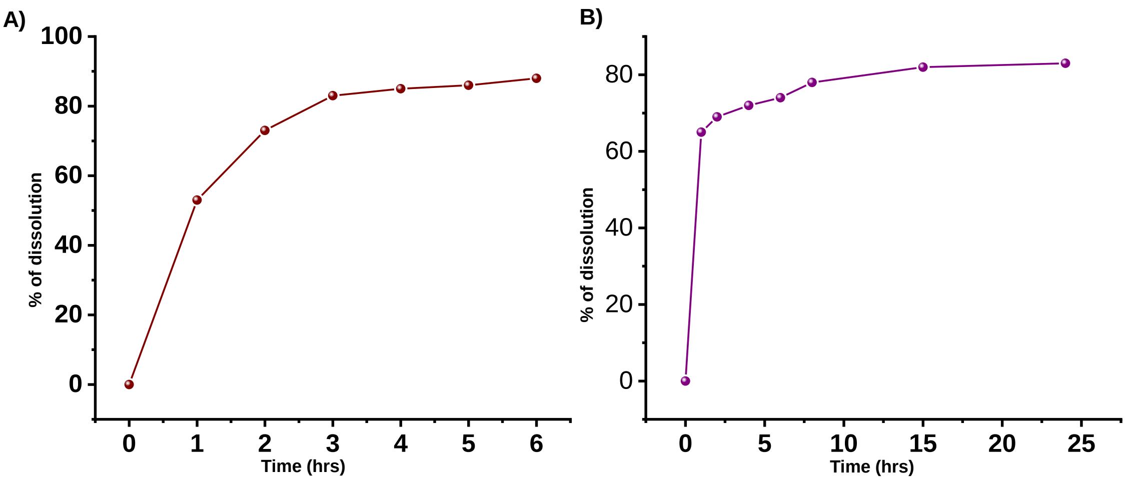

Figure S27. Time-dependent dissolution of Pt for the reaction mixture comprising **A)** 100 mg of fine Pt/C powder (5 wt% Pt) (5 mg, 0.025 mmol), oxalyl chloride (0.45 mmol) and **B)** 100 mg of fine Pt/C powder (5 wt% Pt) (5 mg, 0.025 mmol), oxalyl chloride (0.45 mmol) and H_2O_2 (50 µL, 0.15 mmol) in 3 mL acetonitrile at RT.

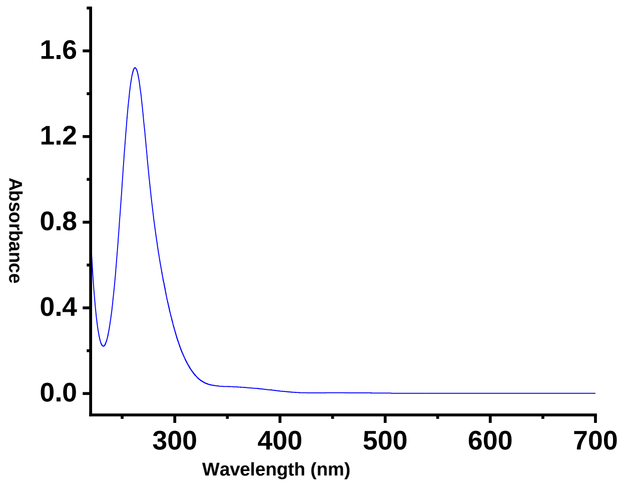

Figure S28. UV-vis spectra of $PtCl_6^{2-}$ after evaporating the leach solution (acetonitrile) and redissolving in 6M HCl.

Entry	Solvent	H_2O_2 (µL)	Oxalyl chloride (mmol)	Time (min)	Dissolution Yield (%)
1	Acetonitrile	50	0.450	2	100

Table S4: The dissolution of Cu in acetonitrile by mixtures of oxalyl chloride and H_2O_2.

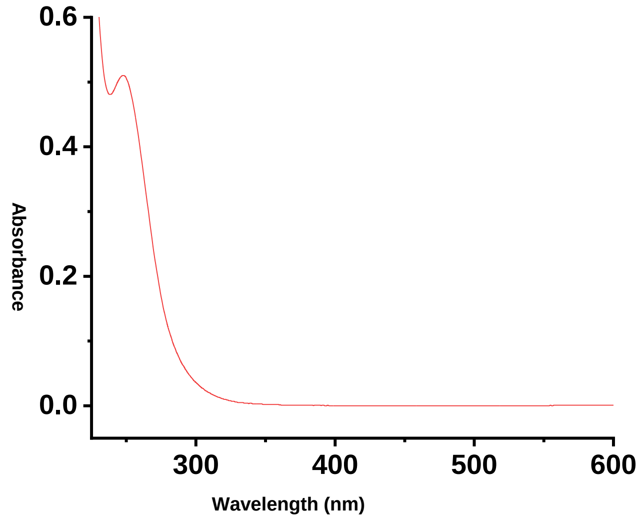

Figure S29. UV-vis spectra of $CuCl_4^{2-}$ after evaporating the leach solution and redissolving in 2M HCl.

References

[1] M. Albayer, J. L. Dutton, *J. Coord. Chem.* **2019**, *72*, 1307-1321.

[2] L. M. M. Kinsman, B. T. Ngwenya, C. A. Morrison, J. B. Love, *Nat. Commun.* **2021**, *12*, 6258.

[3] D. E. James, J. K. Stille, *J. Am. Chem. Soc.* **1976**, *98*, 1810-1823.

[4] D. R. Rowe, W. G. Lloyd, *J. Air. Waste. Manag. Assoc.* **1999**, *49*, 308-315.
